# Biochemical Association of Metabolic Profile and Microbiome in Chronic Pressure Ulcer Wounds

**DOI:** 10.1371/journal.pone.0126735

**Published:** 2015-05-15

**Authors:** Mary Cloud B. Ammons, Kathryn Morrissey, Brian P. Tripet, James T. Van Leuven, Anne Han, Gerald S. Lazarus, Jonathan M. Zenilman, Philip S. Stewart, Garth A. James, Valérie Copié

**Affiliations:** 1 Department of Chemistry and Biochemistry, Montana State University, Bozeman, Montana, United States of America; 2 Center for Biofilm Engineering, Montana State University, Bozeman, Montana, United States of America; 3 Division of Biological Science, University of Montana, Missoula, Montana, United States of America; 4 Department of Dermatology, Johns Hopkins Medical Institutions, Baltimore, Maryland, United States of America; Instituto de Investigación Sanitaria INCLIVA, SPAIN

## Abstract

Chronic, non-healing wounds contribute significantly to the suffering of patients with co-morbidities in the clinical population with mild to severely compromised immune systems. Normal wound healing proceeds through a well-described process. However, in chronic wounds this process seems to become dysregulated at the transition between resolution of inflammation and re-epithelialization. Bioburden in the form of colonizing bacteria is a major contributor to the delayed headlining in chronic wounds such as pressure ulcers. However how the microbiome influences the wound metabolic landscape is unknown. Here, we have used a Systems Biology approach to determine the biochemical associations between the taxonomic and metabolomic profiles of wounds colonized by bacteria. Pressure ulcer biopsies were harvested from primary chronic wounds and bisected into top and bottom sections prior to analysis of microbiome by pyrosequencing and analysis of metabolome using 1H nuclear magnetic resonance (NMR) spectroscopy. Bacterial taxonomy revealed that wounds were colonized predominantly by three main phyla, but differed significantly at the genus level. While taxonomic profiles demonstrated significant variability between wounds, metabolic profiles shared significant similarity based on the depth of the wound biopsy. Biochemical association between taxonomy and metabolic landscape indicated significant wound-to-wound similarity in metabolite enrichment sets and metabolic pathway impacts, especially with regard to amino acid metabolism. To our knowledge, this is the first demonstration of a statistically robust correlation between bacterial colonization and metabolic landscape within the chronic wound environment.

## Introduction

Over the past decade, significant progress has been made in the treatment of acute injuries. However, treatment of chronic wounds such as diabetic foot ulcers, venous leg ulcers, traumatic non-healing wounds, and pressure ulcers remains a major socioeconomic burden with an estimated $58 billion in medical costs associated with the treatment of diabetic ulcers alone [1]. Pressure ulcers in particular are a devastating problem in the elderly and disabled, and an increasing issue of concern in wounded soldiers returning from overseas. Such chronic wounds are defined as lasting greater than 30 days and are characterized by a failure to progress through the normal wound healing process [2]. In healthy, non-immunocompromised individuals, the normal wound healing process initiates quickly and proceeds through well-characterized, iterative steps ranging from hemostasis, inflammation, granulation, epithelialization, and maturation [3]. In chronic wounds the major contributing factor to wound persistence is bioburden in the form of colonizing bacteria [4].

The metagenome of the human-colonized microbiome, consisting of species commensal and pathogenic, is 100 times larger than the human genome [5], with the four most dominant bacterial phyla being Firmicutes, Bacteroidetes, Proteobacteria, and Actinobacteria. However, distribution of these four phyla varies significantly from tissue to tissue. For example, Firmicutes and Bacteroidetes are the most abundant phyla in the gastrointestinal tract [5, 6]. Proteobacteria is the most abundant phyla in the airway [7], and Proteobacteria and Actinobacteria dominate the skin environment [8]. Recent characterization of colonizing bacteria in diabetic versus non-diabetic wounds in mouse models demonstrated a shift in bacterial microbiota concurrent with impaired wound healing [9]. These data suggest that culture-based methods of characterizing wound microbiota has historically underestimated the diversity of species present and highlights a need for identification of organisms through non-culture-based methods [10]. Within the environment of a chronic wound, significant diversity of bacterial microbiota has been demonstrated [11], and likely varies across the changing landscape of the wound based on nutrient availability and nutritional gradients. Indeed, the bacterial microbiota profile of the wound is likely directly related to depth of sampling with increased abundance of anaerobic bacteria in areas of low oxygen availability, as anaerobic bacteria are commonly found in chronic wounds [12].

Within the mucosa, the profile of the bacterial microbiota is shaped by metabolic, immunological, secretory, and structural changes [5, 6, 8, 13]. However, less is known about factors that influence bacterial growth and community in the wound environment. Bacteria exist in multiple physiological states including viable, dormant, and non-viable and pathogenic potential is likely to differ according to distinct physiological state [14], with the currently accepted model being that highly metabolically active bacteria are more pathogenic, and more metabolically dormant bacteria contribute to wound persistence. It is thus of particular interest to understand how the metabolic environment of the wound contributes to the make-up of the bacterial communities that colonize a chronic wound.

Systems biology utilizes the integration of multiple “omics” approaches to create a comprehensive representation of a biological system, and is a key driver towards the ultimate goal of developing protocols for personalized medicine [15]. Global profiling of small metabolites (referred to as metabolomics) holds significant promise as a tool for personalized medicine as it provides a close read-out of the phenotypic characteristics of a given biological system. NMR is particularly adept for global metabolite profiling in biological systems as profiles can be obtained from crude samples [16] and direct identification and quantification of metabolites can be obtained[17]. While metabolomics is a nascent field with novel diagnostic applications, characterization of the metabolic environment holds promise for developing personalized treatment plans that favor beneficial colonization by bacteria, pathogen clearance, and progress towards wound healing. With this ultimate goal in mind, in this pilot study we demonstrate the integration of bacterial metagenomic profiling with metabolomics to identify correlations between bacterial community composition and metabolic environment within the landscape of the chronic wound.

## Methods

### Ethics Statement

Clinical data was collected at the time of sample procurement (Table 1) and Montana State University Institutional Review Board approval was obtained (Protocol #GJ030107). Written informed consent was obtained and Subject identification was blinded at sampling.

**Table 1:** Details of clinical subjects and chronic pressure ulcer wounds.

	Patient Number			
	1	2	3	4
**Patient Details**				
**Age**	63	27	23	43
**Gender**	Male	Female	Female	Male
**Race**	African American	African American	White, non-Hispanic	African American
**Comorbidities**	None	CP*, MR^ŧ^, scoliosis, seizure disorder	DVT^§^, anemia, bipolar, HSV^¶^	UTI**, anxiety, depression
**Gross Nutritional Status**	Well-nourished	G-tube fed, liquid only, malnourished	Well-nourished	Inadequate protein intake
**Clinical Presentation**				
**Cause**	Paralysis	ICU stay	Lower thoracic paraplegia	Paralysis
**Location**	Left hip	Left hip	Right ischium	Left ischium
**Duration**	2 years	7 months	3 years	1.5 years
**Length (cm)**	2	3	2	5.5
**Width (cm)**	1.1	1.5	2.4	5
**Depth (cm)**	0.1	1.5	2.5	3
**Systemic Antibiotic**	Bactrim	None	Vancomycin	Doxycyline
**Topical Antibiotics**	None	None	None	Mesalt
**Bacterial culture before biopsy**	Acinetobacter, Corynebacterium, Staphylococcus aureus	Not done	Corynebacterium negative for anaerobes	Not done

*CP = cerebral palsy,

^ŧ^MR = mental retardation,

^§^DVT = deep vein thrombosis,

^¶^HSV = herpes simplex virus,

** = urinary tract infection

### Sample Collection of Pressure Ulcer Biopsies

Chronic wound samples were collected as described previously [18]. Briefly, pressure ulcer biopsies were collected from four patients presenting to the Johns Hopkins Wound Center. Chronicity was defined as wounds lasting greater than 30 days [2]. Biopsy samples were flash frozen in liquid nitrogen, shipped via dry ice, and stored at -80°C prior to taxonomy and metabolomics analysis.

### Bacterial DNA Extraction, Amplification, and Sequencing

Chronic wound samples were thawed and cut into small pieces with a clean razor. DNA was extracted according to the standard MoBio Power BiofilmDNA Isolation Kit procedures. For each sample, we amplified a ~500bp portion of the 16S rRNA gene using the 16S Eubacterial primers in S1 Table. PCR reactions were set up in a PCR hood using DNA/RNA free reagents according to manufacturer’s protocol (Promega GoTaq Green Master Mix) and cleaned with QIAquick PCR Purification columns. The amplicons were visualized on a 1% agarose gel stained with ethidium bromide. PCR products were quantified with Promega's QuantiFluor dsDNA System and pooled in an equimolar ratio. A non-template control (NTC) was included for each primer set, then pooled equivolume, and spiked into the sequencing pool. Pyrosequencing was conducted at Engencore at the University of South Carolina on a 454 Life Sciences Genome Sequencer FLX instrument (Roche) using titanium chemistry. The 16S rRNA sequences were processed and taxonomically binned using the QIIME pipeline [19]. Sequences were de-multiplexed and quality-filtered using default QIIME settings. Chimeric reads were identified and removed using chimeraslayer and reads matching sequences from the NTC using exclude_seqs_by_blast.py with 98% identity and 1E-100 e-value cutoff values. OTU clustering was done at the 97% similarity threshold and taxonomic identities were assigned using RDP classifier [20]. QIIME was used to generate taxonomy plots using summarize_taxa_through_plots.py.

#### Multivariate Data Analysis

Two-dimensional principal component analysis (2D-PCA) was performed as described previously [17]. Briefly, samples were classified according to whether the sample originated from the top or bottom section of the wound biopsy and clustered by PCA according to relative bacterial phyla bioburden using XLSTAT version 3.1 software (Addensoft) and Pearson correlation. 2D-PCA accounted for 73.14% of the total variance using principal components 1 and 2 to generate the scores plot. Factor loadings for all seven principal components of the analysis are shown in S3 Table. A threshold of significance for correlation coefficients was set at >0.400, as used previously [17].

### NMR Compound Identification and Quantification

#### Metabolite Extraction

Small molecule metabolites were extracted as described previously [17] with some modifications. Biopsy samples were resuspended in ice-cold 60% aqueous methanol and homogenized using a tissue homogenizer (Tissue Tearor Model 985370–395, Biospec Products Inc., Bartlesville, OK) set to 2-minute intervals of 10 seconds on and 5 seconds off. Tissues were transferred to glass tubes and lysed by sonication prior to addition of 1:1 aqueous choloroform and vortexing. Aqueous layers were collected by centrifugation and transferred to clean tubes prior to lyophilization for 4 hours with low heat. Lyophilized samples were resuspended in 550 μL of NMR buffer (10mM NaH_2_PO_4_/ Na_2_HPO_4_ containing 0.5 mM 4,4-dimethyl-4-silapentane-1-sulfonic acid (DSS) in 100% D_2_O, pH 7), assayed for pH, and transferred to 5mm NMR tubes (Bruker) prior to analysis.

#### NMR Analysis

1D ^1^H NMR spectra was acquired as described previously [17]. NMR spectra were acquired on a Bruker 600-MHz (1H Larmor frequency) AVANCE III solution NMR spectrometer equipped with a SampleJet automatic sample loading system, a 5 mm triple resonance (1H, 15N, 13C) liquid helium-cooled TCI probe (cryoprobe), and Topspin software (Bruker version 3). One-dimensional NMR spectra were acquired using the Bruker supplied noesypr1d pulse sequence with 256 scans, using a spectral width of 9600 Hz at 298K (25°C). Free induction decays were collected into 32K data points, with a dwell time interval of 52 μsec amounting to an acquisition time of ~ 1.7 sec, using a 2 second relaxation recovery delay between acquisitions, and a NOESY mixing time period of 50 msec. Spectral processing and analysis was performed using the Chenomx NMR software (version 6.0) (Chenomx Inc., Edmonton, AB, Canada) according to recommended protocols and previous metabolomics analyses [17, 21, 22]. For each sample, NMR spectra were manually phased, baseline corrected, a line broadening function of 0.5 Hz applied, and calibrated to DSS at δ = 0.0 ppm. For metabolite identification, the Chenomx small molecule library for 600-MHz (1H Larmor frequency) magnetic field strength NMR was used, and NMR spectral patterns were fitted for each sample independently. The internal DSS standard was used for quantitation of identified metabolites.

#### Univariate and Multivariate Data Analysis

Quantified metabolite concentrations (see S5 Table) were normalized to tissue mass prior to chemometric analysis. Tissue-mass normalized metabolite concentrations were then scaled and log transformed to obtain data values with a Gaussian distribution to avoid systemic bias in multivariate analysis. Data distribution before and after normalization is shown in S1 Fig.

For univariate analysis, the 25 most significant metabolites were first identified by t-test (significance threshold p-value < 0.05) then used to generate a correlation heatmap identifying metabolites that cluster according to location of biopsy section (top or bottom). Distance measure was generated by Pearson correlations and clustering using the Ward algorithm. Significantly contributing metabolites to univariate analysis were determined using volcano plots, which identify significant features by combining t-test (p < 0.05) and fold change analysis (FC > 2) thresholds. Box-whisker plots graphically display the relative concentrations of significant metabolites following normalization of the quantified NMR data.

Multivariate data analysis was conducted by using the supervised classification method three-dimensional partial least square-discriminant analysis (3D PLS-DA). 3D PLS-DA accounted for 49.60% of the total variability in the first three principal components. The number of latent variables in the model was determined by the cross-validated sum of the squares captured by the model (*Q* ^*2*^) and data over fitting was detected using permutation tests [23]. Significantly contributing metabolites were identified using the variable importance in projection (VIP), which was calculated on the weighted sum of the squares for the partial least squares (PLS) loadings.

#### Metabolite Set Enrichment and Reconstruction of Metabolic Pathways

Metabolite set enrichment analysis (MSEA) is based on the more commonly used gene set enrichment and identifies groups of metabolites that associate with biological functions or metabolic pathways [24, 25]. Functional grouping of metabolites was determined using the quantitative enrichment analysis (QEA) algorithm based on the globaltest algorithm [26]. For each metabolite set associated with a pathway, the total metabolites, profiled metabolites, Holm P value, and false discovery rate (FDR) is shown (S6 Table).

Global metabolic pathway analysis was performed using topological data analysis, allowing complex dimensional reconstruction of the putative metabolic pathways impacted from low dimensional data. Topological data analysis is a recently applied application to the mining of large data sets [27]. Topology analysis estimates the importance of a metabolite within a metabolic network by estimating the compound’s degree and betweenness centrality, thus calculating the contribution of the compound to the global metabolic network based on the number of edges and amount of control exerted by each node on neighboring nodes. Node color and size are indicative of p-value significance and pathway impact, respectively.

#### Correlation of Taxonomy and Metabolite Profiles

The nonparametric Spearman rank correlation was used to quantify the association between the relative bacterial load and the metabolite profiles of the top and bottom sections of the pressure ulcer wound biopsies. Correlations were determined for all phyla detected at least as 0.1% of the total bacterial load (data not shown), and all genera for the three most abundance phyla with a relative bacterial load greater than 0.1%. Correlations were considered significant for correlation coefficient threshold values greater than 0.700 and p-value greater than 0.05.

## Results

### Pressure ulcer bacterial microbiome predominated by three main phyla: Firmicutes, Proteobacteria, and Actinobacteria

**Fig 1:**
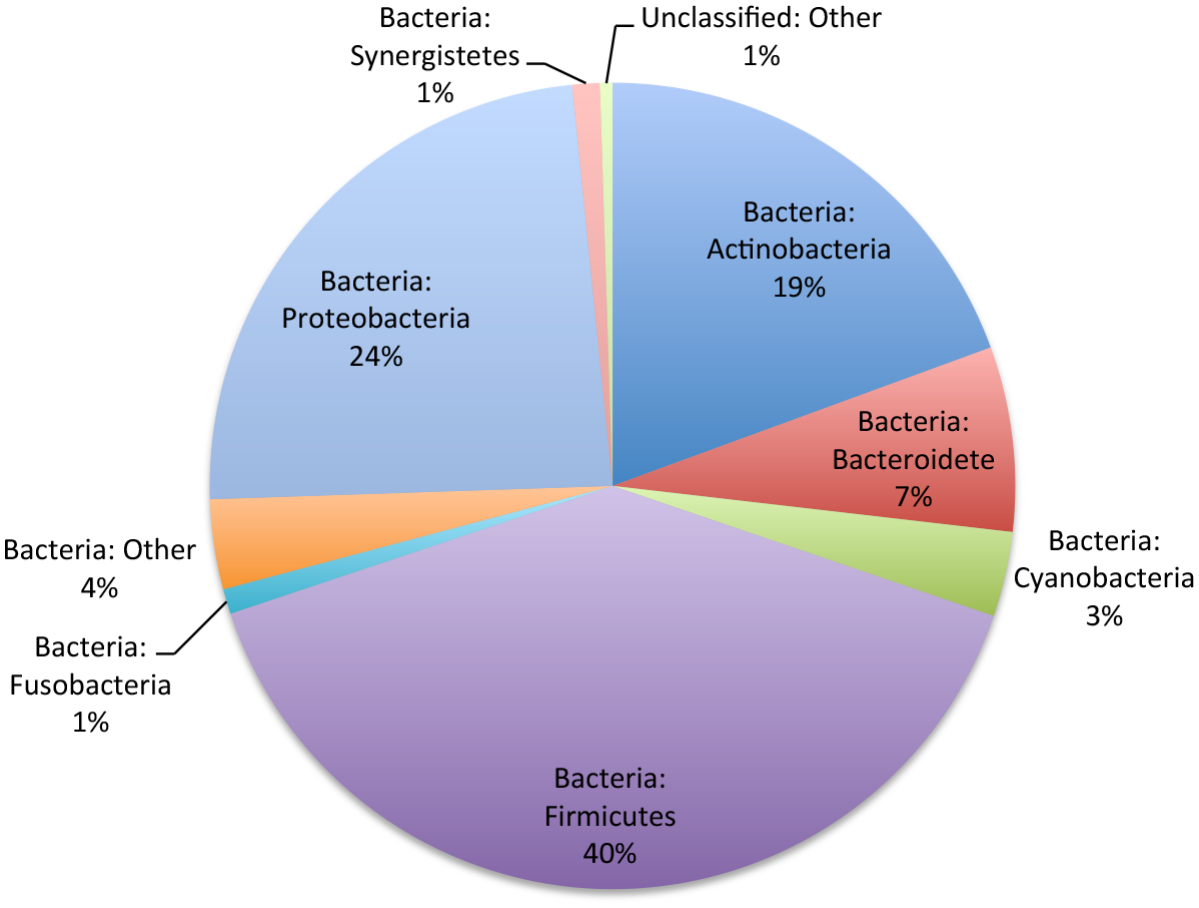
Mean abundance of major bacterial phyla for samples (n = 8) from clinical pressure ulcer wounds (n = 4).

The bacterial load within the patient pressure ulcers was determined by pyrosequencing the variable regions 1–2 of eubacterial 16S rRNA genes from punch biopsy samples. Overall the pressure ulcers were colonized by three predominant bacterial phyla: Firmicutes (40%), Proteobacteria (24%), and Actinobateria (19%). To a lesser extent, Bacteroidetes (7%), Cyanobacteria (3%), Fusobacteria (1%), and Synergistes (1%) were also detected. Remaining sequences were assigned to low abundance (<0.1%) OTUs or unclassified OTUs (5%) (Fig 1).

**Fig 2:**
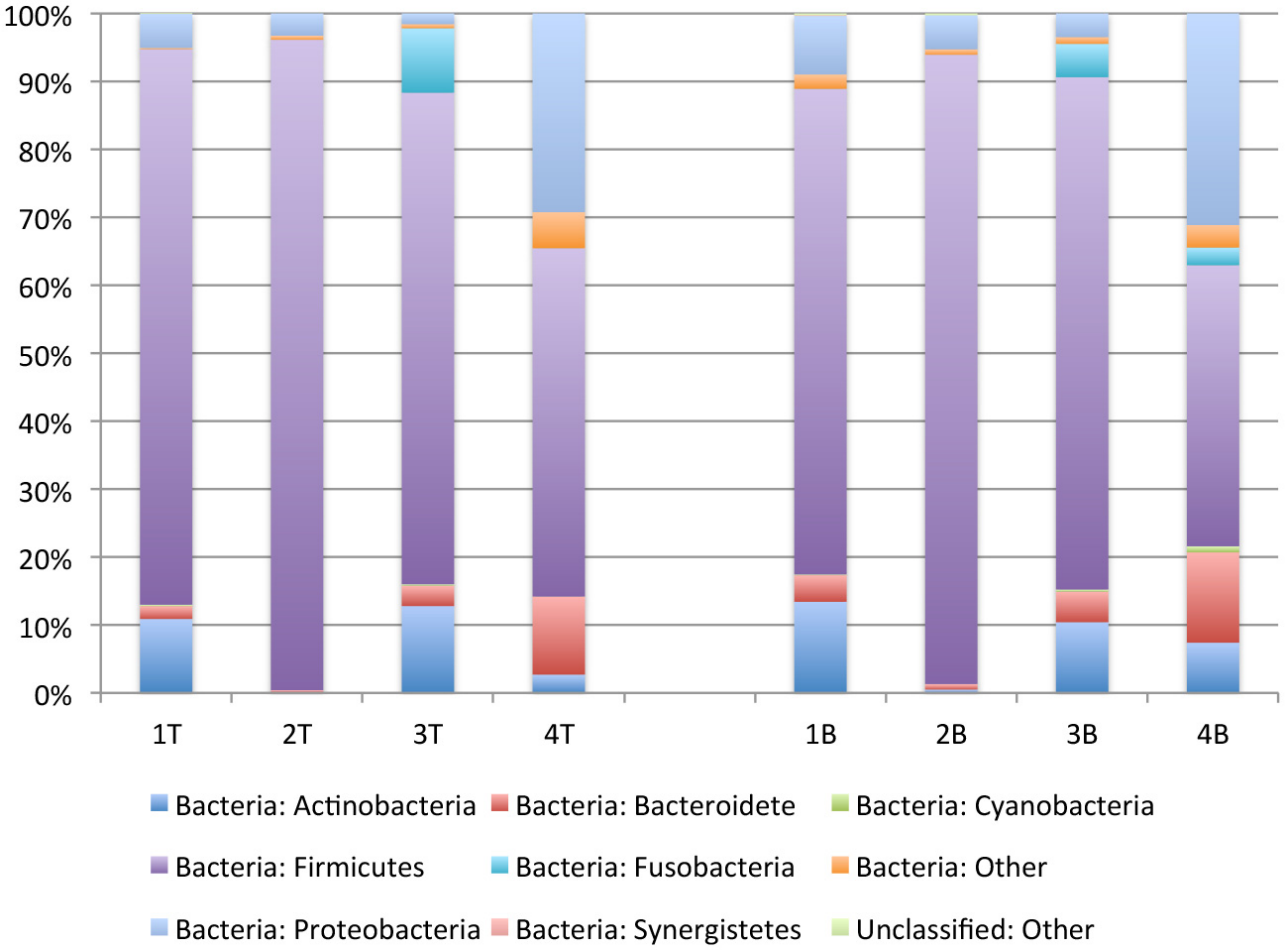
Relative abundance of major bacterial phyla for chronic pressure ulcer biopsy samples harvested from the top (T) and bottom (B) of the wounds.

Within each wound, the dominant phyla remained consistent between the top and bottom sections of the biopsy; however, there was variation between samples from different wounds (Fig 2 and S2 Table). Firmicutes were the most abundant phylum sequenced from all samples, varying in relative abundance from 95.7% in the top section of the biopsy from Subject two (Figs 1 and 2T) to 41.4% in the bottom section of the biopsy from Subject four (Figs 1 and 4B). Across all samples, Firmicutes was relatively more abundant in the top section of the wound biopsy than in the bottom section suggesting that the bacterial load from this phylum varies across the landscape of the wound.

The second most abundant phylum also varied across subjects with Proteobacteria being the second most abundant for Subject four (29.3% and 31.2%, 4T and 4B, respectively) and Subject two (3.3% and 5.0%, 2T and 2B, respectively). For Subjects one and three, the second most abundant phylum was Actinobacteria (10.9% and 13.4%, 1T and 1B, respectively; 12.8% and 10.4%, 3T and 3B, respectively). For the phyla detected at a lesser relative abundance, Bacteroidetes were predominantly identified from Subject four in both top and bottom sections of the wound biopsy (11.5% and 13.3%, 4T and 4B, respectively). Cyanobacteria were identified in the top and bottom biopsy sections of Subject one (0.2% and 0.1%, 1T and 1B, respectively) and three (0.2% and 0.3%, 3T and 3B, respectively), and in the bottom biopsy section of Subject four (0.9%, 4B). Lastly, Bacteroidetes were identified in all subjects, but were most abundant in the top and bottom biopsy sections of Subject four (11.5% and 13.3%, 4T and 4B, respectively) (Fig 2 and S2 Table).

**Fig 3:**
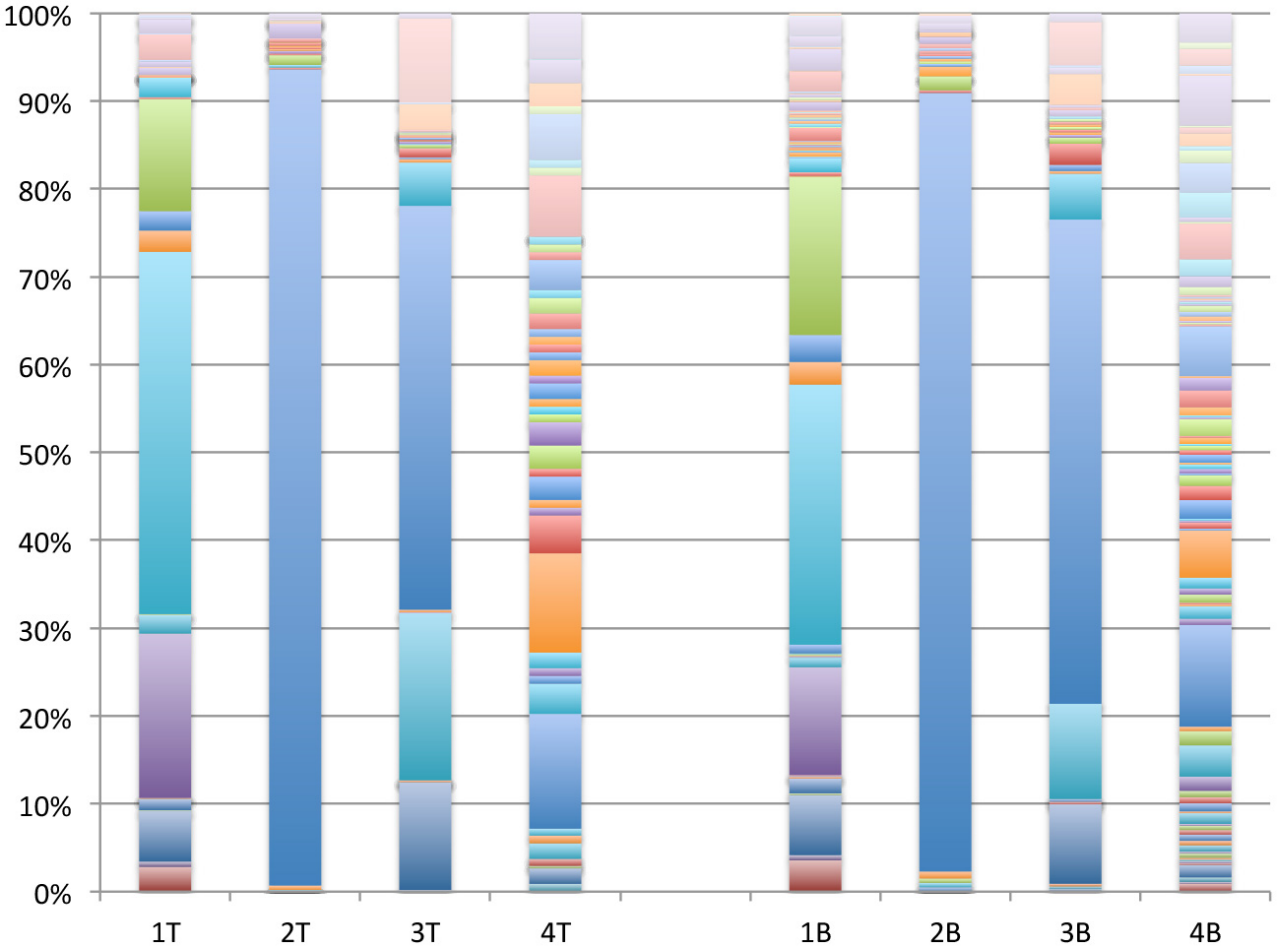
Relative abundance of major bacterial genera for chronic pressure ulcer biopsy samples harvested from the top (T) and bottom (B) of the wounds.

**Fig 4:**
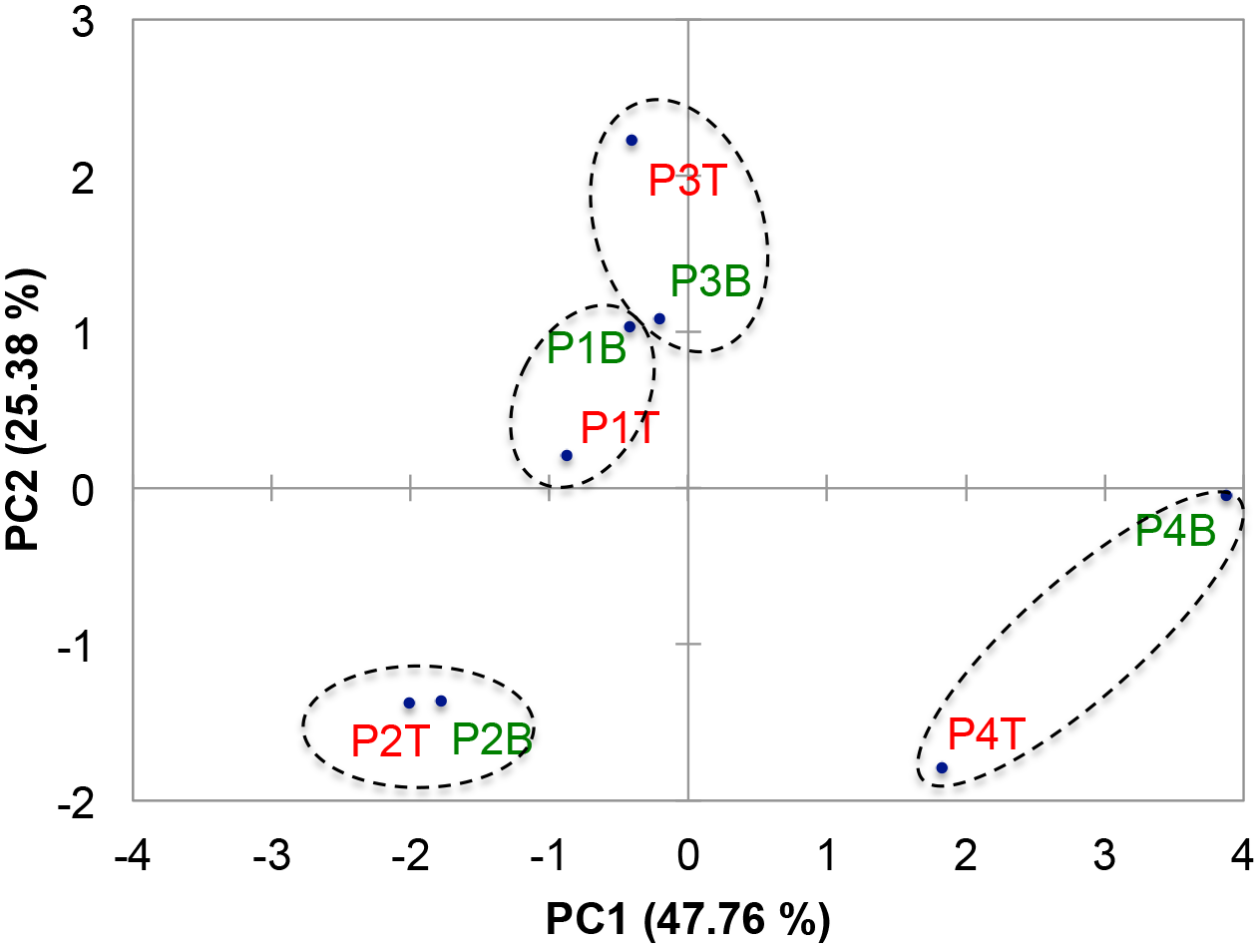
Two dimensional principal component analysis (2D PCA) scores plot demonstrates statistical clustering of top (T) and bottom (B) biopsies of the same wounds (n = 4) rather than statistical clustering dependent on biopsy depth based on analysis of relative bacterial abundance.

While at the phylum-level the bacterial microbiome within the pressure ulcers appears fairly similar, further analysis of the genera that comprised a relative abundance of at least 0.1% demonstrates significant variability across samples with substantial variability between wounds and limited variability within wounds (Fig 3 and S3 Table). Present in all samples, *Streptococcus* varies from 93.1% in the top section of the biopsy from subject two (Figs 3 and 2T) to less than 1.0% in the bottom section of the biopsy from subject one (Figs 3 and 1B). Despite being from the top of a relatively shallow wound sample the bacterial microbiome for phylum Firmicutes sequenced from subject one was dominated by two Gram-positive anaerobic cocci *Peptoniphilus* and *Anaeroccocus* and a Gram-positive facultative anaerobe *Gemella* (Fig 1 [1T and 1B] and S3 Table). In comparison, the deeper bottom wound profiled from subject four (Figs 3, 4T and 4B), had a high relative abundance of the obligate anaerobe class Clostridia of phylum Firmicutes (33.8% and 16.2%, 4T and 4B, respectively), represented predominantly by the common digestive tract colonizer family *Lachnospiraceae* (11.5% and 5.3%, 4T and 4B, respectively). Interestingly, while *Staphylococcus* is an opportunistic pathogen from phylum Firmicutes commonly isolated from chronic wounds using culture-based methods, as seen for subject one (see Table 1), metagenomic profiling identified a high relative abundance of *Staphylococcus* only in the biopsy from subject three (18.9% and 10.8%, Fig 3, 3T and 3B, respectively).

In the overall analysis of bacterial load, the pressure ulcers were colonized by a relative abundance of 24% phylum Proteobacteria and 19% phylum Actinobacteria (Fig 1). However, sequence from subject four contributed to the substantial weight of the bacterial load from Proteobacteria with a relative abundance of 29.3% and 31.2%, top and bottom of the wound, respectively (Figs 2, 4T and 4B, respectively). More in-depth analysis indicates that the predominant contributors to phylum Proteobacteria in these wound samples are of the genus *Burkholderia* (10.7% and 13.5%, 4T and 4B, respectively) and *Pseudomonas* (7.1% and 8.2%, 4T and 4B, respectively). Phylum Actinobacteria was predominantly sequenced from wound biopsies taken from subjects one and three, with relatively equal abundance from the top of the wound, at 10.9% and 12.8% (1T and 3T, respectively), and the bottom of the wound, at 13.4% and 10.4% (1B and 3B, respectively) (Fig 2). The predominant genera contributors to the bacterial load from phylum Actinobacteria included *Actinomyces* in subject one (2.8% and 3.6%, 1T and 1B, respectively) and *Corynebacterium* in both subject one (5.7% and 6.9%, 1T and 1B, respectively) and subject three (12.2% and 9.0%, 3T and 3B, respectively) (Fig 3 and S3 Table).

To determine whether wound biopsies could be statistically distinguished based on bacterial bioburden, we utilized two-dimensional principal component analysis (2D PCA) of the relative abundance of each detected phylum to statistically cluster samples based on their microbiome. Despite high representation of the phylum Firmicutes across all samples, PCA demonstrated statistical separation of samples from subjects one, two, and three (1T/1B, 2T/2B, 3T/3B, respectively) from subject four (4T/4B) along principal component one (PC1) and statistical separation of samples from subjects one and three (1T/1B and 3T/3B, respectively) from subjects two and four (2T/2B and 4T/4B) along principal component two (PC2) (Fig 4). Statistically significant contributing variables to separation along PC1 included the relative abundance of phyla Bacteroidetes, Cyanobacteria, Firmicutes, and Proteobacteria, while statically significant contributing variables to separation along PC2 included relative abundance of Actinobacteria and Fusobacteria based on weight of correlation coefficients (S4 Table). Interestingly, samples clustered within the same quadrant of the PCA scores plot regardless of whether the biopsy section was from the top or bottom of the wound. This suggests that bacterial bioburden varies more significantly between wounds rather than within the depth of the wound, despite the fact that all of the wounds are classified as chronic pressure ulcers. These findings indicate that while phyla Firmicutes, Proteobacteria, and Actinobacteria contribute substantially to the relative bacterial bioburden within these pressure ulcer wounds, there is substantial genus variation between wounds including obligate aerobes and facultative and obligate anaerobes.

### NMR Metabolomics of Chronic Pressure Ulcer Biopsies Identifies Distinguishable Metabolite Profiles Based on Depth of Biopsy Section

To determine whether the metabolic environment of chronic pressure ulcers changed across the depth of the wound, we sectioned wound biopsies into top and bottom samples and performed 1D ^1^H NMR profiling of small molecule metabolites (>3KDa) extracted from these wound samples. NMR spectral features were identified, quantified, and assigned to specific metabolite IDs using the Chenomx library of small molecules for 600 MHz NMR spectrometers, and absolute metabolite concentrations were normalized to tissue mass. Of the over 300 metabolite compounds contained in the Chenomx metabolite database, 122 metabolites were detected in at least one sample from one subject wound biopsy in the top and/or bottom biopsy sections (see S5 Table). Quantified metabolite concentrations spanned several orders of magnitude. In order to reduce statistical bias from more abundant metabolites and therefore misidentification of significant variable contributing to the sample profiles, the data were normalized by log transformation and scaled (mean-centered and divided by the standard deviation of each variable) to obtain a Gaussian distribution. The concentration distribution of the 50 most significantly differentiated metabolites detected are shown in S1 Fig both before (left panel) and after (right panel) data normalization. Kernel density plots below the list of metabolites indicates how normalization transformed the data set into a Gaussian distribution prior to statistical analysis.

**Fig 5:**
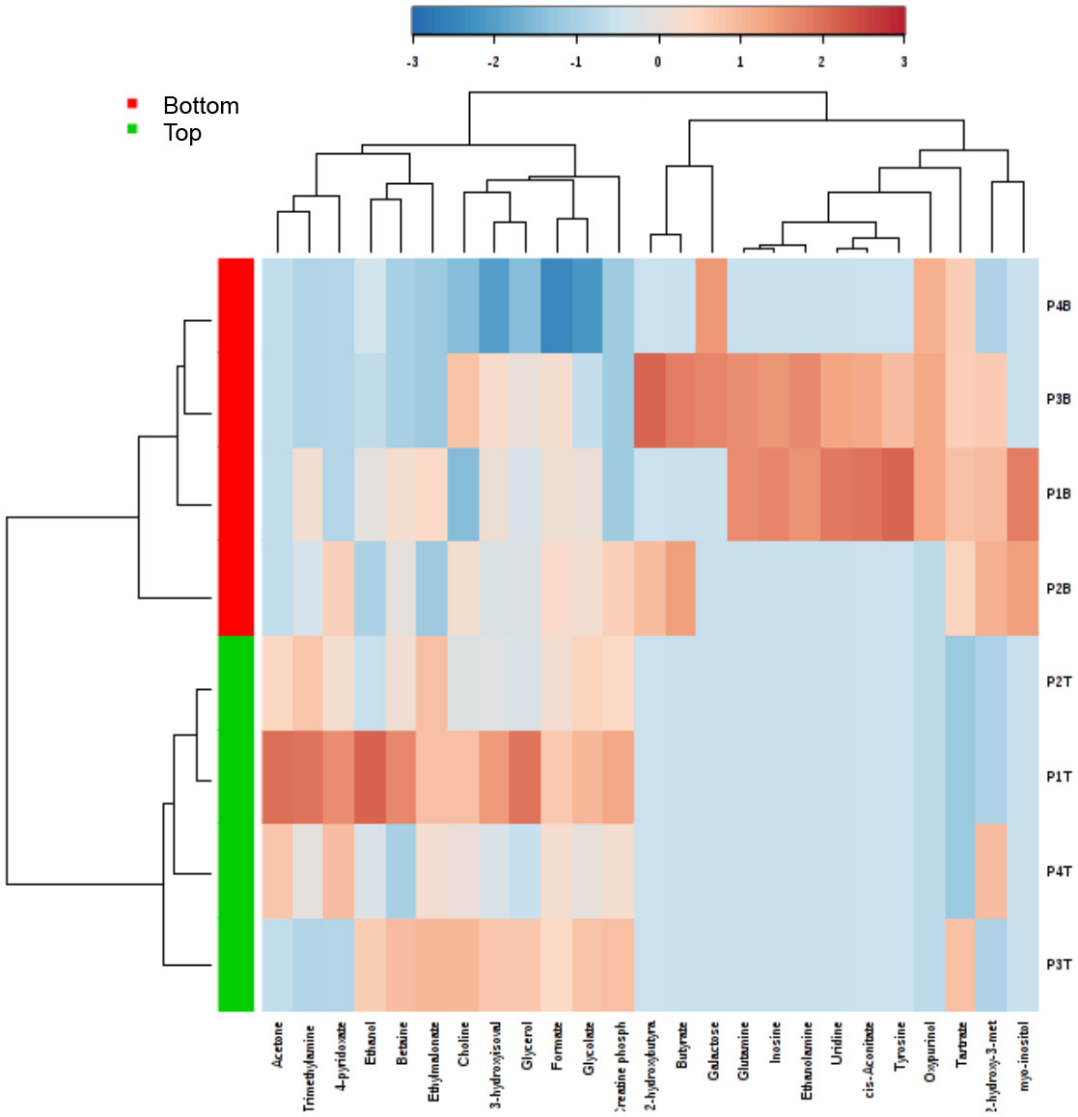
Heatmap visualization. Heatmap was constructed based on clustering results from metabolite profiles of chronic pressure ulcer biopsies sectioned into top (green) and bottom (red) samples. Heatmap features the top twenty-five metabolite features as identified by t-test analysis (p≤0.05). Distance measure is by Pearson correlation and clustering is determined using the Ward algorithm.

**Fig 6:**
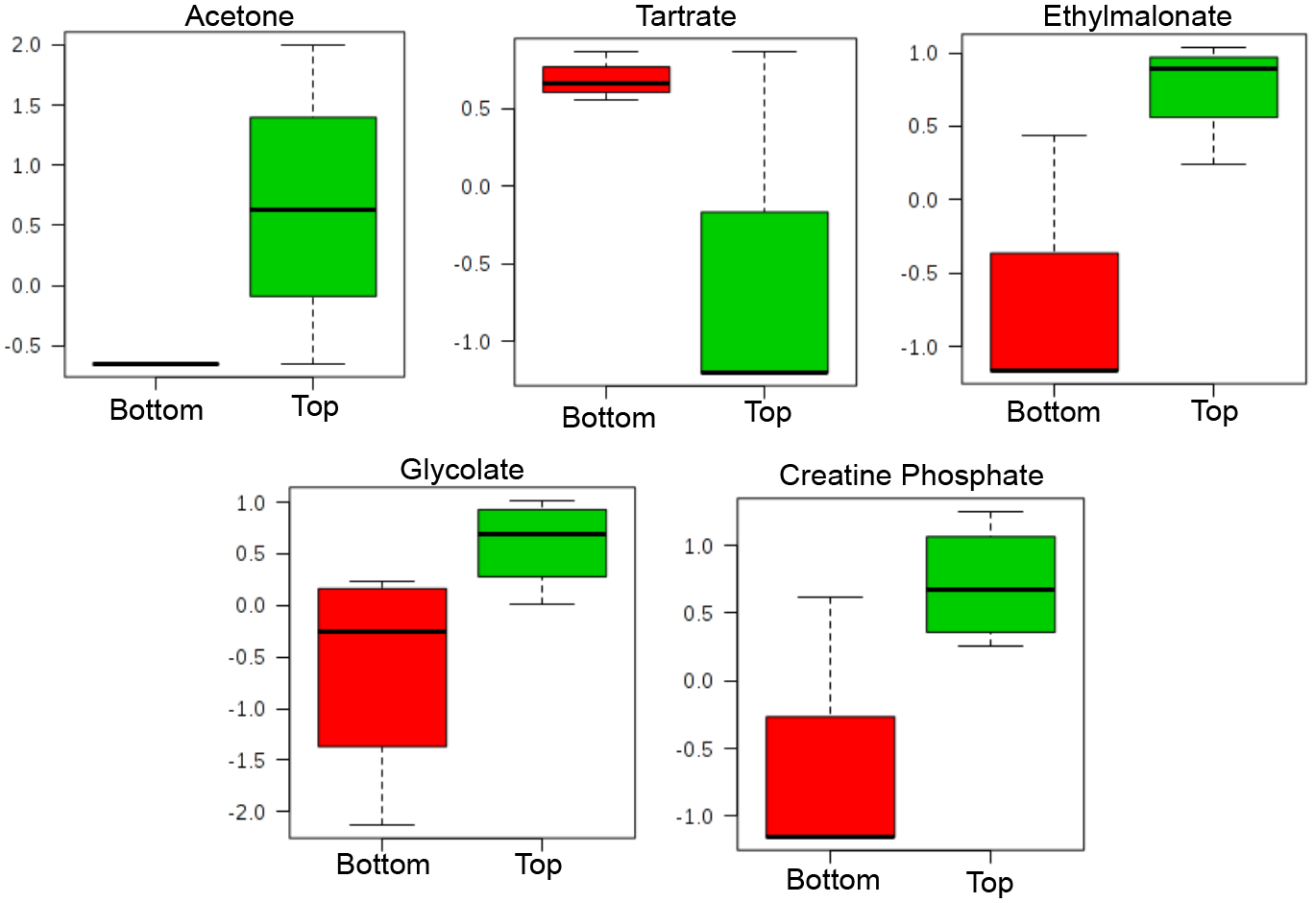
Box-whisker plot graphs of metabolites with significant concentration differences profiled from top and bottom sections of chronic pressure ulcer wounds. Important metabolites were selected by volcano plot which is a combination of fold change analysis (FC≥2.0) and t-test analysis (p≤0.05). Box-whisker plots are calculated from normalized concentrations (y-axis).

Using both univariate and multivariate statistical analyses, the metabolite profiles were used to separate samples according to depth of biopsy sections. Despite the variability in wound depth (Table 1), the biopsy samples separated into top and bottom sections across all pressure ulcer samples in a statistically robust manner (Figs 2 and 3). Heatmap visualization of the clustering of metabolite profiles based on the 25 most significant metabolites as identified by t-test analysis (p≤0.05) demonstrated statistically significant correlation between all sections originating from the top wound biopsies and separately, from the bottom wound biopsies (Fig 5). Metabolites that contributed significantly to the distinction between top and bottom wound sections included increased relative concentration of ethylmalonate, creatine phosphate, and glycolate in top sections, decreased relative concentration of acetone in bottom sections, and increased relative concentration of tartrate in bottom sections of the wound biopsies (Fig 6). Given the low number of subjects available to us for this pilot study, we also statistically analyzed the significance of these contributing metabolites by the significance analysis of microarray (SAM) algorithm [28], which addresses false discovery rate by running iterative tests. For a delta threshold of 0.1, ethylmalonate, creatine phosphate, glycolate, tartrate, and acetone were all identified as significantly contributing variables to the clustering of metabolite profiles based on whether the section originated from the top or the bottom of the biopsy sample.

**Fig 7:**
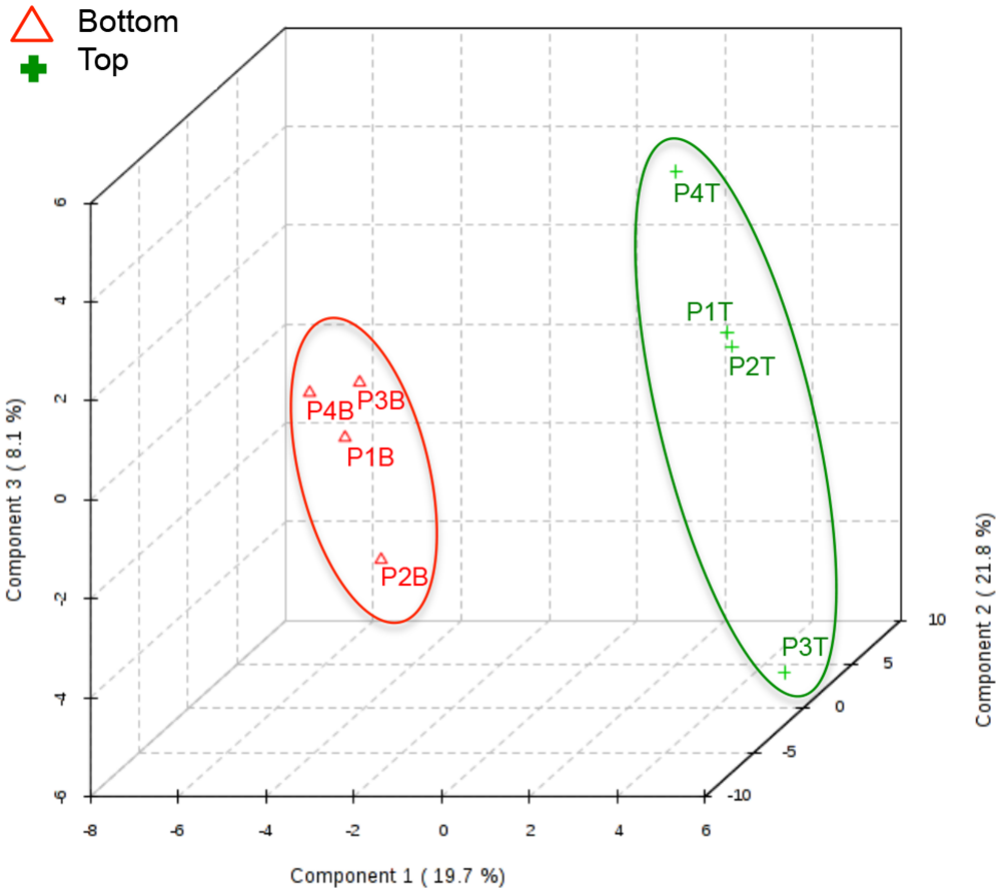
Scores plot of 3D PLS-DA statistically clusters chronic wound samples based on depth of biopsy. Red triangles indicates sections from the bottom of the wound biopsy and green crosses indicates sections from the top of the wound biopsy. 49.6% of the variance observed in the matrix of metabolite profiles is explained by the first 3 components.

**Fig 8:**
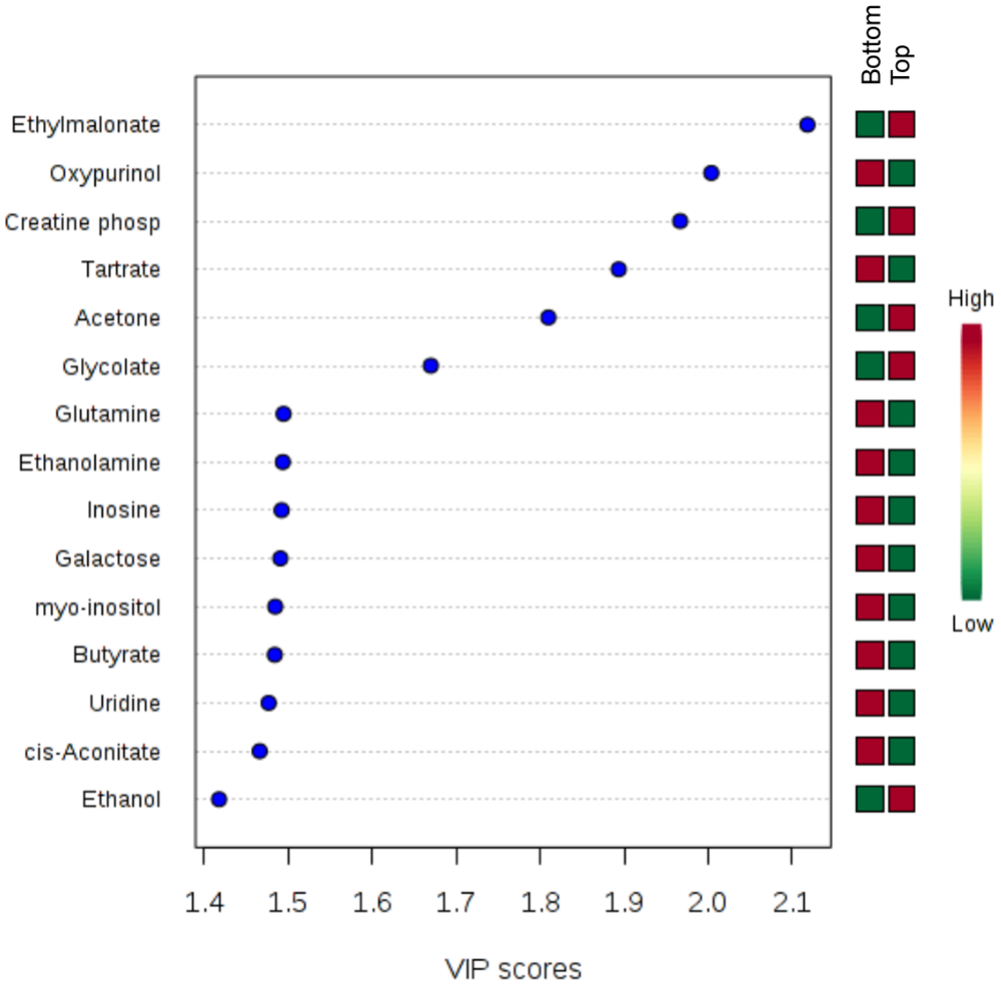
Variable importance in projection (VIP) plot displays the top 15 most important metabolite features identified by PLS-DA. Colored boxes on right indicate relative concentration of corresponding metabolite for samples biopsied from the bottom and top of chronic wounds. VIP is a weighted sum of squares of the PLS-DA loadings taking into account the amount of explained Y-variable in each dimension.

Because of the high-dimensionality of metabolomics data, multivariate statistical analysis is well suited for determination of statistically significant changes in metabolite concentrations across the complex environmental landscape of a pressure ulcer biopsy. The normalized metabolite concentrations were thus subjected to partial least squares—discriminant analysis (PLS-DA), a supervised regression technique for classifying groups from multidimensional data. Three dimensional PLS-DA (3D PLS-DA) of the metabolite profiles for the biopsy sections showed clear separation of samples based on the depth of the biopsy section with bottom sections clustering closer together (red triangles) than top sections (green crosses) (Fig 7). The three principal components used to generate the scores plot accounted for almost 50% of the variance across the data set, with ~ 20% of the variance contributing to the separation between the top and bottom biopsy sections along the first PC dimension (Fig 7). Contribution of the variables was assessed by examining the variable importance in projection (VIP) score, which is calculated from the weighted sum of the square for each PLS loadings for each component as described in [29]. Of the top fifteen variables identified by VIP scores, ethylmalonate, creatine phosphate, tartrate, acetone, and glycolate were all identified as metabolite variables that significantly contributed to the class separation of the top and bottom wound biopsy sections based on characteristic metabolite profiles (Fig 8). Taken together, the univariate and multivariate statistical analyses demonstrate that, despite significant variability in microbial bioburden between wounds, metabolic variability within a wound is conserved from wound to wound, as established based on metabolite profiles of top and bottom sections of biopsies.

### Metabolic Pathway Mapping Indicates Significant Impact on Selective Amino Acid Metabolism within the Chronic Pressure Ulcer Environment

**Fig 9:**
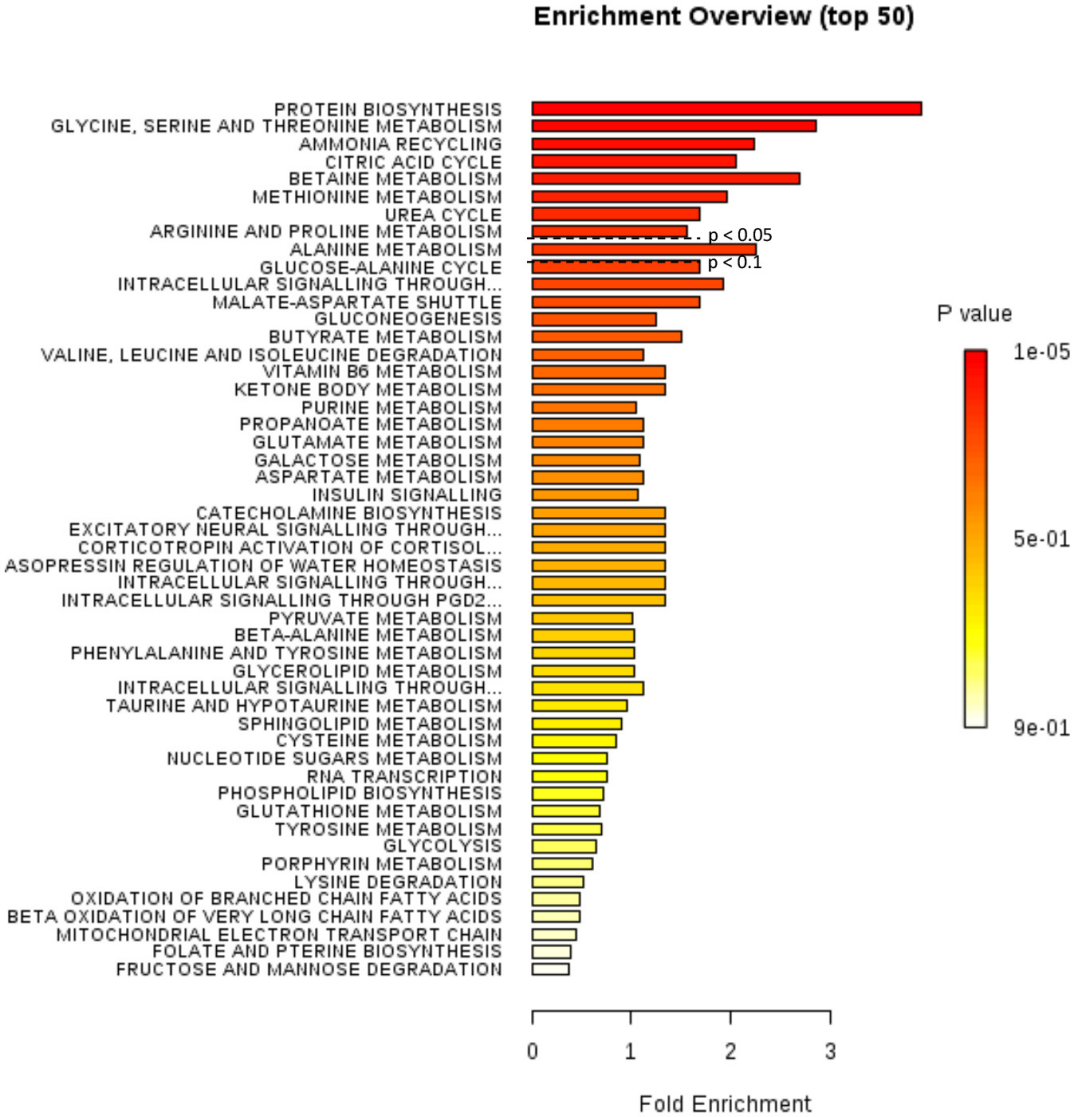
Metabolic pathway analysis demonstrated major impact in amino acid metabolism. Summary plot for metabolite set enrichment analysis (MSEA) where metabolite sets are ranked according to p-value with hatched lines indicating Holm p-value threshold. The top fifty metabolite sets are shown. The color code of the bar plot corresponds to the calculated *P* values.

Metabolite profiling demonstrated a clear separation between the top and bottom sections of the wound biopsies. To further investigate the association between these discrete sets of metabolic markers, we performed metabolite set enrichment analysis (MSEA) using the quantitative enrichment analysis (QEA) based on the globaltest algorithm [26]. This algorithm utilizes a linear model to estimate the association between metabolite concentration profiles and the sample class. Because of the clear separation between top and bottom sections of the wound biopsies, we used “top of wound biopsy” and “bottom of wound biopsy” as our class identification. In QEA-MSEA, the p-value indicates the strength of the association between the metabolite profiled and the class label. Of the 50 most significant metabolites identified (Fig 9), eight global metabolic processes were found significant (p<0.1, FDR<1%), including protein biosynthesis, amino acid metabolism, ammonia recycling, the citric acid cycle (TCA), and the urea cycle (Fig 9 and S6 Table). These data indicated that metabolite profiles from top and bottom sections of the wound biopsies not only represent discrete sets of small molecule biomarkers, but that these biomarkers can be organized into cohesive, statistically robust metabolite parameters characteristic of coordinated activation of metabolic pathways.

**Fig 10:**
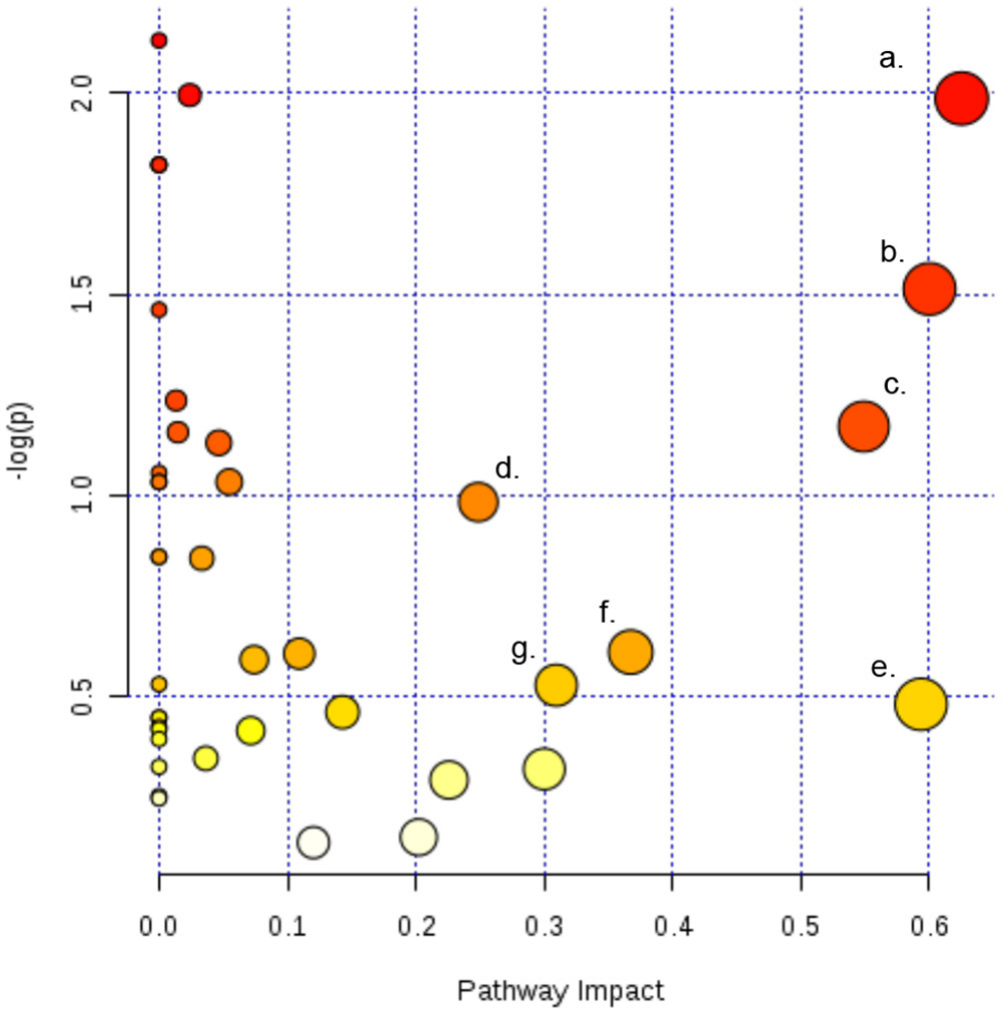
Putative metabolic pathways associated with the wound environment of chronic pressure ulcers. Metabolome summary of pathway analysis. Mapping of the relative concentration of metabolites to the metabolome indicates impact contribution of metabolic pathways. Node color indicates significance based on p-value and node size indicates significance of pathway impact. Significantly impacted pathways include (a) inositol phosphate metabolism, (b) glyoxylate and dicarboxylate metabolism, (c) alanine, aspartate, and glutamate metabolism, (d) arginine and proline metabolism, (e) glycine, serine, and threonine metabolism, (f) pyruvate metabolism, and (g) citric acid cycle (TCA). The metabolic pathways are arranged according to the scores from the enrichment analysis (Fig 4) (Y-axis) and from the topology analysis (X-axis).

To more comprehensively decipher how top and bottom sections of the wound biopsies result in distinct metabolic activity patterns, the MetPA software was used to identify specific metabolic pathways associated with the small molecule profiles identified by NMR (Fig 10). Topology analysis estimates the importance of a metabolite within a metabolic network. Node color and size are indicative of p-value significance and pathway impact, respectively. Based on this analysis, the pathways most impacted by biopsy depth include (a) inositol phosphate metabolism, (b) glyoxylate and dicarboxylate metabolism, (c) alanine, aspartate, and glutamate metabolism, (d) arginine and proline metabolism, (e) glycine, serine, and threonine metabolism, (f) pyruvate metabolism, and (g) citric acid cycle (TCA) (Fig 10).

**Fig 11:**
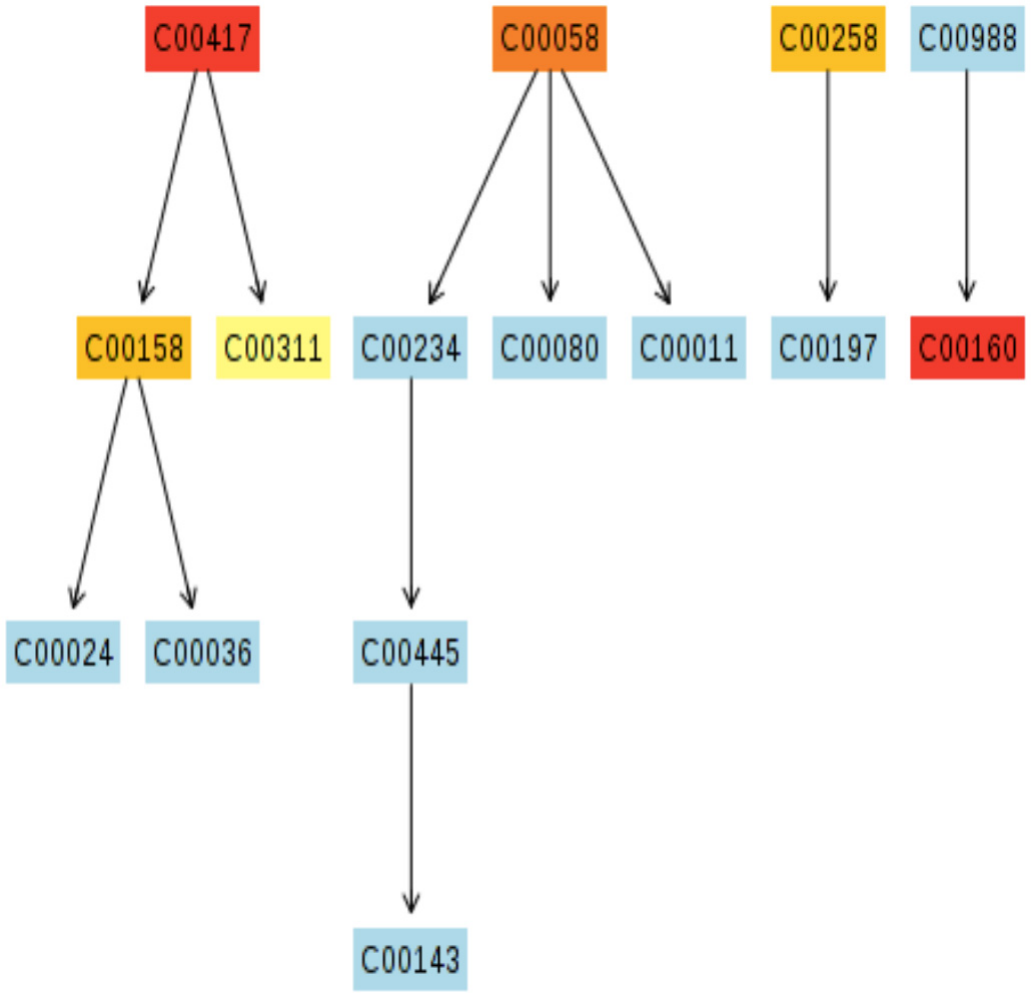
Putative metabolic pathways associated with the wound environment of chronic pressure ulcers. Significantly contributing pathway nodes include glyoxylate and dicarboxylate metabolism. Highlighted metabolites indicated hits from the metabolic profiling and are coded according to p-value. Pathway maps are generated using the KEGG reference map (http://www.kegg.jp/kegg/pathway.html).

**Fig 12:**
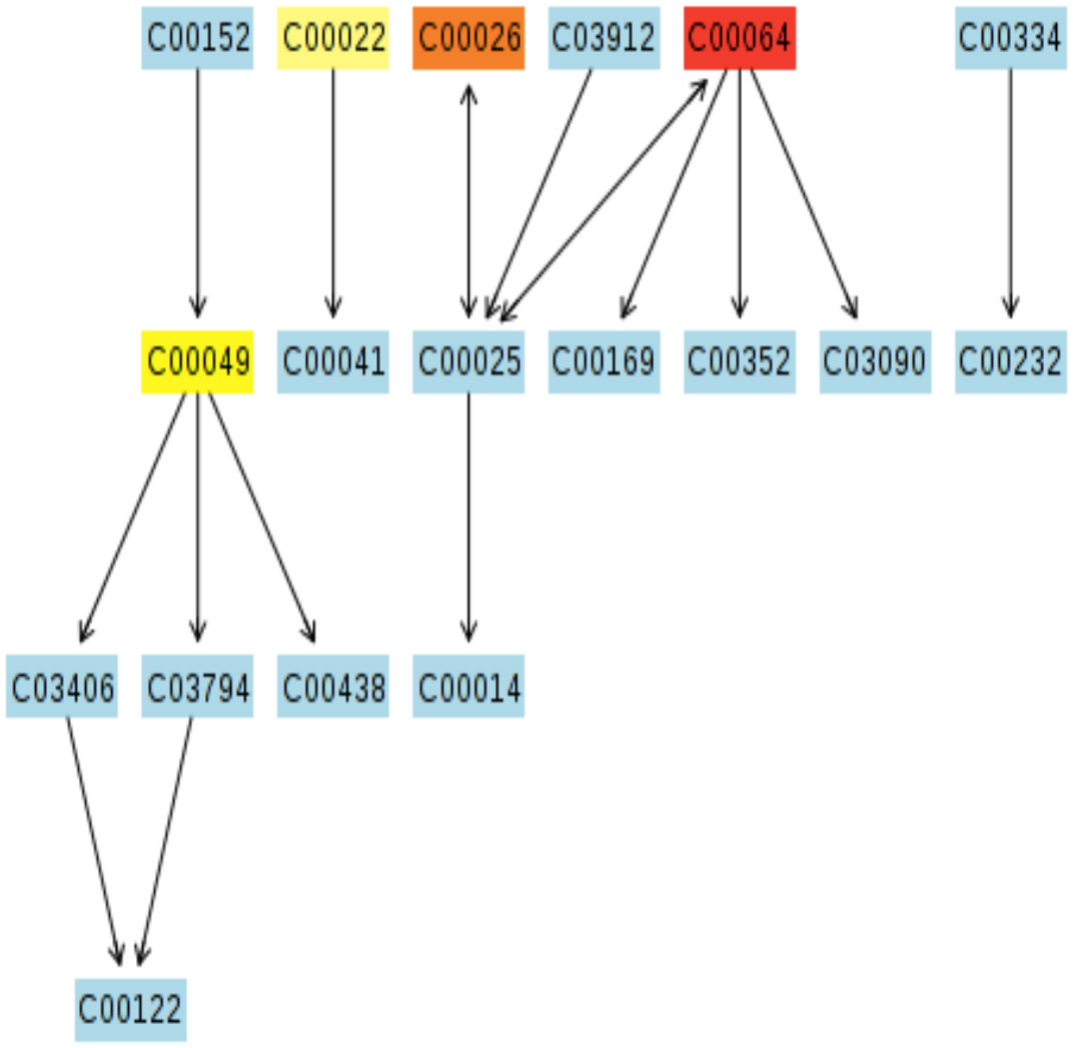
Putative metabolic pathways associated with the wound environment of chronic pressure ulcers. Significantly contributing pathway nodes include alanine, aspartate, and glutamate metabolism. Highlighted metabolites indicated hits from the metabolic profiling and are coded according to p-value. Pathway maps are generated using the KEGG reference map (http://www.kegg.jp/kegg/pathway.html).

**Fig 13:**
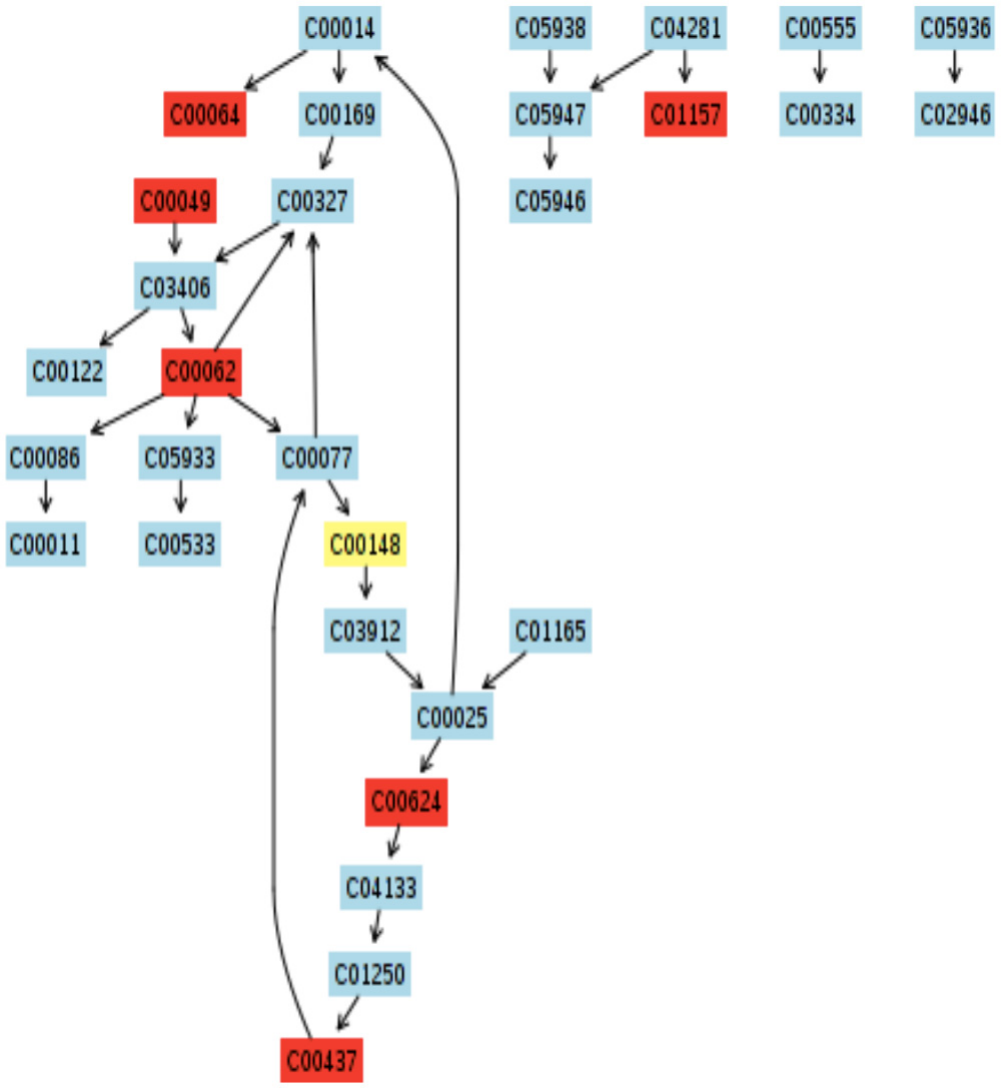
Putative metabolic pathways associated with the wound environment of chronic pressure ulcers. Significantly contributing pathway nodes arginine and proline metabolism. Highlighted metabolites indicated hits from the metabolic profiling and are coded according to p-value. Pathway maps are generated using the KEGG reference map (http://www.kegg.jp/kegg/pathway.html).

**Fig 14:**
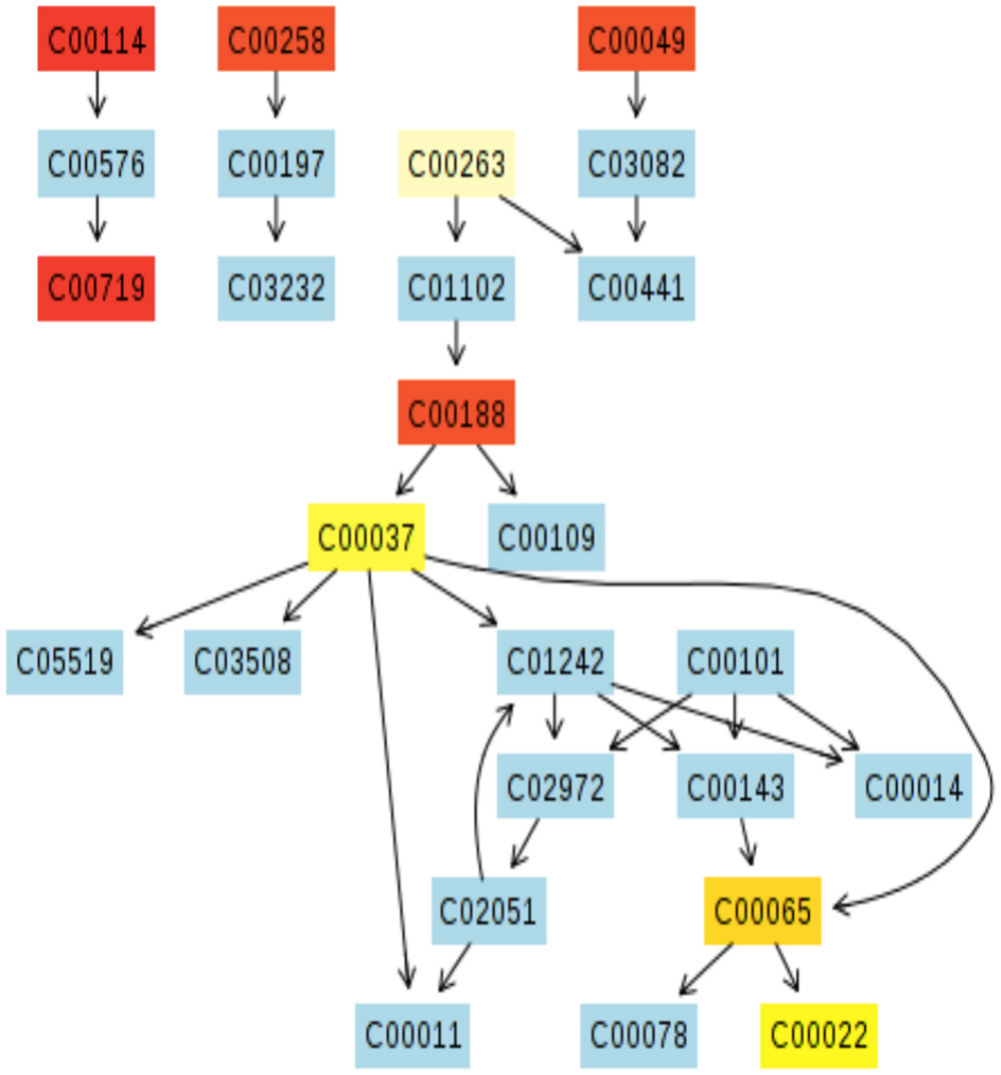
Putative metabolic pathways associated with the wound environment of chronic pressure ulcers. Significantly contributing pathway nodes include glycine, serine, and threonine metabolism. Highlighted metabolites indicated hits from the metabolic profiling and are coded according to p-value. Pathway maps are generated using the KEGG reference map (http://www.kegg.jp/kegg/pathway.html).

Metabolic pathway visualization using the KEGG database indicates compound contribution to pathways of particular interest (Figs 11–14). While the MetPA determined 22 pathways significantly impacted when comparing top and bottom sections of wound biopsies (Fig 10), strongly impacted pathway nodes included glycolate and dicarboxylate metabolism (Fig 11); alanine, aspartate, and glutamate metabolism (Fig 12); arginine and proline metabolism (Fig 13); and glycine, serine, and threonine metabolism (Fig 14). Metabolite contributions to these pathway nodes also overlapped. For example, glycerate concentration profiles significantly impacted glycolate and dicarboxylate metabolism (p<0.1), and glycine, serine, and threonine metabolism (p<0.01) (Figs 11 and 14). Aspartate concentration profiles significantly impacted alanine, aspartate, and glutamate metabolism (p<0.05); arginine and proline metabolism (p<0.01), and glycine, serine, and threonine metabolism (p<0.01). Glutamine concentration profiles significantly impacted alanine, aspartate, and glutamate metabolism (p<0.01), and arginine and proline metabolism (p<0.01). Finally, pyruvate concentration profiles were found to significantly impact alanine, aspartate, and glutamate metabolism (p<0.05), and glycine, serine, and threonine metabolism (p<0.05). Both the MSEA and metabolic pathway impact analysis indicated that target pathways for metabolic activity within the chronic pressure ulcer environment are associated with selective amino acid metabolism.

### Correlation Between Bacterial Microbome and Metabolite Profiles within the Chronic Pressure Ulcer Environment

**Fig 15:**
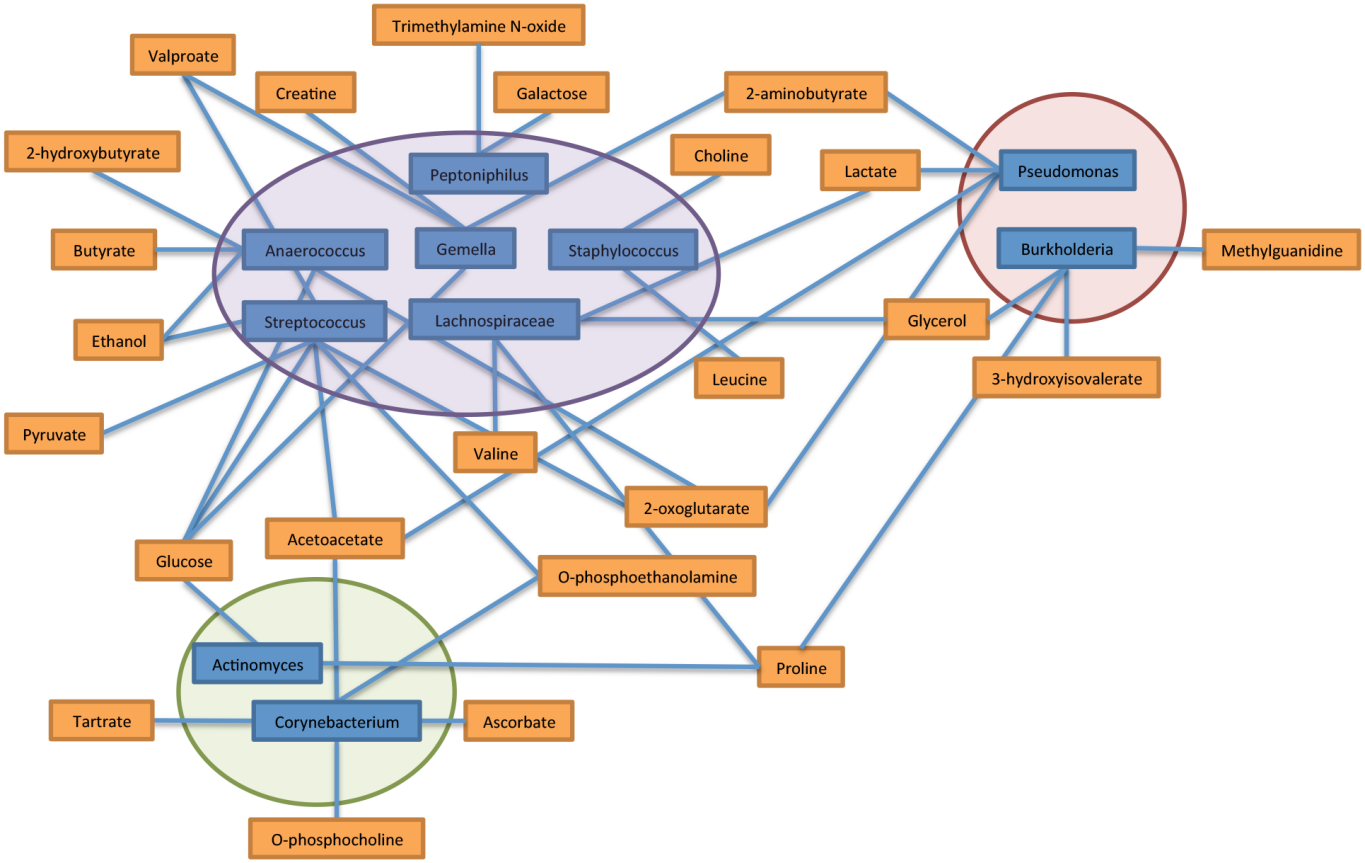
Correlation between the bacterial microbiome and metabolome in chronic pressure ulcer wounds. Nonparametric Spearman rank correlation was used to quantify the association between the relative abundance of bacterial genera and metabolite concentration in chronic pressure ulcer wounds. Major genera observed across wound samples are shown (see Fig 3) with phylum Firmicutes (purple), phylum Proteobacteria (red), and phylum Actinobacteria (green) clustered together. Correlation coefficient threshold of significance is set at 0.700 and p-values ≤ 0.05.

Finally, we investigated the possible link between the taxonomic profiles and small molecule metabolite profiles distinguishing top and bottom sections of the chronic pressure ulcer biopsies. The relative abundance of 10 genera from the three phyla accounting for the majority population detected by 16S rRNA sequence were correlated to normalized metabolite concentrations. The nonparametric Spearman rank correlations of genera and metabolite profiles from top and bottom biopsy sections displayed significant correlation between each genus and multiple metabolites based on correlation coefficients (threshold of >0.700, p-value <0.05) (Fig 15).

Of the most significant metabolites, changes in glucose concentration correlated significantly with the most commonly sequenced genera including the Firmicutes genera *Anaerococcus*, *Streptococcus*, and *Gemella* (purple shading) and the Actinobacteria genus *Actinomyces* (green shading). A selective number of amino acids were found to correlate with the presence of specific genera including proline with *Actinomyces* and *Burkhoderia*, leucine with *Staphylococcus*, and valine with *Lachnospiraceae*. The ketone bodies acetoacetate and 2-hydroxybutyrate were found to be associated with *Streptococcus*/*Corynebacterium* and *Anaerococcus*, respectively. Two metabolites commonly associated with gut flora metabolism butyrate and trimethylamine N-oxide (TMNO) correlated significantly with two Gram-positive anaerobic cocci *Anaerococcus* and *Peptoniphilus*, respectively. In combination with the MSEA, metabolic pathway impact analysis, and multivariate statistical analysis, this correlation analysis suggests that while taxonomic profiles are distinct between wounds and metabolic profiles are similar when comparing depth of wound biopsy, there may be unique metabolite expression patterns indicative of specific genera colonizing the chronic pressure ulcer wound.

## Discussion

In this exploratory study, we have demonstrated an association between the colonizing microbiota and metabolic landscape of chronic, pressure ulcer wounds. Although this demonstration study included only a small number of patient subjects, our statistically robust findings lend credibility to this approach as a useful means to assess the complexity of host-pathogen interactions within a chronic wound environment. After sectioning wound biopsies into top and bottom sections, we determined that each sample was colonized by three main phyla, but that there was significant variability at the genus level. While the taxonomic profiling demonstrated unique relative contributions of bacterial genera to wound bioburden between wounds, metabolite profiling demonstrated significant similarity in the metabolic landscape, specifically for pathways associated with amino acid metabolism. To our knowledge, this is the first demonstration of a statistically robust correlation between bacterial colonization and metabolic landscape within the chronic wound environment.

Despite nearly two decades of pyrosequencing, there remains little consensus regarding the predominant organisms contributing to bacterial bioburden within a chronic wound. While Firmicutes and Bacteroidetes are the most abundant phyla in the gastrointestinal tract [5, 6], Proteobacteria is the most abundant phyla in the airway [7], and Proteobacteria and Actinobacteria dominating the skin environment [8], we found all four of these phyla represented in our wound biopsy samples, with the predominant presence of Firmicutes, Proteobacteria, and Actinobacteria. Culture methods used for bacterial identification have long demonstrated that *Staphylococcus* and *Pseudomonas* are predominant organisms in chronic wounds [30]. However, more recent pyrosequencing methods have indicated that the reported predominance of these organisms is likely the result of ease of culturing rather than the true representation in the wound environment. Indeed, in agreement with previous taxonomic profiles [11], we found *Peptinophilus*, *Streptococcus*, and *Clostridium* to be significant contributors to wound bacterial bioburden. Despite these similarities, wound colonization by bacteria varies significantly from wound to wound (even in our small sample size) and taxonomic profiles alone may not provide sufficient insights into foundational functional mechanisms at the root of wound chronicity.

In contrast to the multivariate analysis of the taxonomic profiles, we could establish significant correlation between wound samples based on the depth of the biopsy section and small molecule metabolite profiles. For example, significant changes were found in acetone, creatine phosphate, tartrate, glycolate, and ethylmalonate using multiple statistical techniques, and these metabolites were associated with metabolic pathways impacted by metabolite set enrichment such as glycine, serine, threonine metabolism, alanine, aspartate, and glutamate metabolism, and arginine and proline metabolism. Interestingly, these amino acid metabolic pathways have also been associated with differentiation between the biofilm and planktonic phenotype in *Staphylococcus aureus* and *Pseudonomas aeruginosa* ([17] & manuscript in preparation).

Functional association between the bacterial “who” and the “what are they doing” of the colonized wound has the potential to uncover key insights into the complexity of host-pathogen interactions within the chronic wound environment, and may provide potential avenues for therapeutic intervention. For example, we identified an association between the facultative anaerobe *Cornebacterium* and the dicarboxylic acid tartrate. Uptake and degradation of tartrate by *Cornebacterium* provides enhanced metabolic flexibility in anaerobic and low pH environments allowing this resident of the human gut to pathogenically colonize the female genital tract [31]. Interfering with such metabolic strategies may become a low-cost means of targeting this wound colonizer by creating a metabolically hostile environment. For example, we found an association between *Pseudomonas* and lactate metabolism and *Burkholderia* and glycerol metabolism. By targeting these alternative carbon sources, therapeutic interventions could hinder the competitiveness of these opportunistic pathogens [32–34].

The observation that alternate carbon sources are correlated to the presence of specific bacteria may result from metabolic strategies employed by either, or both, the host and the pathogen, as any trauma to the skin results in metabolic perturbation at the wound site including mobilization of amino acids from muscle and gluconeogenesis.[35] Therapeutic interventions might benefit well from a better understanding of the functional relationship between the localized microflora and metabolic landscape of the wound. For example, we found arginine metabolism impacted in our pressure ulcer samples, as well as a metabolic marker differentially impacted in the biofilm mode of growth for both *S*. *aureus* and *P*. *aeruginosa* [17] (manuscript in preparation) and oral arginine supplementation enhanced wound healing in both healthy young adults and the elderly [36–38]. While malnutrition is known to contribute to delays in wound healing and predisposition to development of a chronic wound[39–41], and the nutritional status of the subjects was known (Table 1), the effect nutrition has at the wound site is outside of the scope of this study.

A major goal of the NIH Human Microbiome Project is to determine how shifts in the microbiome relate to health and disease with the ultimate goal of improving health through monitoring and manipulating the microbiome [42]. Understanding biochemical associations between colonizing microbes and the metabolic landscape not only provides key insight into complex host-pathogen interactions, but also potentially identifies microbial features and metabolomic signatures that could be used as diagnostic biomarkers to identify clinical populations at risk for wound infection. Insights into these biological connections hold the potential for non-invasive methods for diagnostic and targeted therapeutic interventions. Finally, through targeted manipulation of the metabolic environment, therapeutic interventions could potentially shift the wound microbiome to inhibit pathogenic bacteria and promote colonization by symbiotic bacteria. While these goals are outside of the scope of this current study, we have demonstrated in this exploratory research the potential for identifying interconnections between the metabolic landscape and the colonizing microbiome of chronic wounds that could be exploited for diagnostic, prognostic, and therapeutic potential.

## Supporting Information

S1 Fig Mean variation in metabolite concentration. Box plots and kernel density plots before and after normalization are shown with boxplots indicating top 50 metabolite features and density plots based on all metabolites detected. Selection method for normalization was log transformation with column-wise scaling in which each concentration was mean-centered and divided by the standard deviation of each variable.
(TIF)

S1 Table 16S eubacterial primers used for 16S rRNA gene amplification. (DOCX)

S2 Table Relative abundance (%) of bacterial phyla in chronic pressure ulcers. (DOCX)

S3 Table Relative abundance (%) of bacterial genera in chronic pressure ulcers. (XLSX)

S4 Table Factor loadings for 2D PCA of relative bacterial abundance in chronic pressure ulcers. (DOCX)

S5 Table Metabolites detected by ^1^H NMR in chronic pressure ulcer wounds. (XLSX)

S6 Table Summary of metabolite set enrichment analysis (MSEA) ranked by *P* value (p≤0.1) (DOCX)
