# Supplementary figures and images for: Biochemical Association of Metabolic Profile and Microbiome in Chronic Pressure Ulcer Wounds
Source: PLoS One. 2015 May 15;10(5):e0126735. doi: 10.1371/journal.pone.0126735 (PMC4433261; doi:10.1371/journal.pone.0126735)

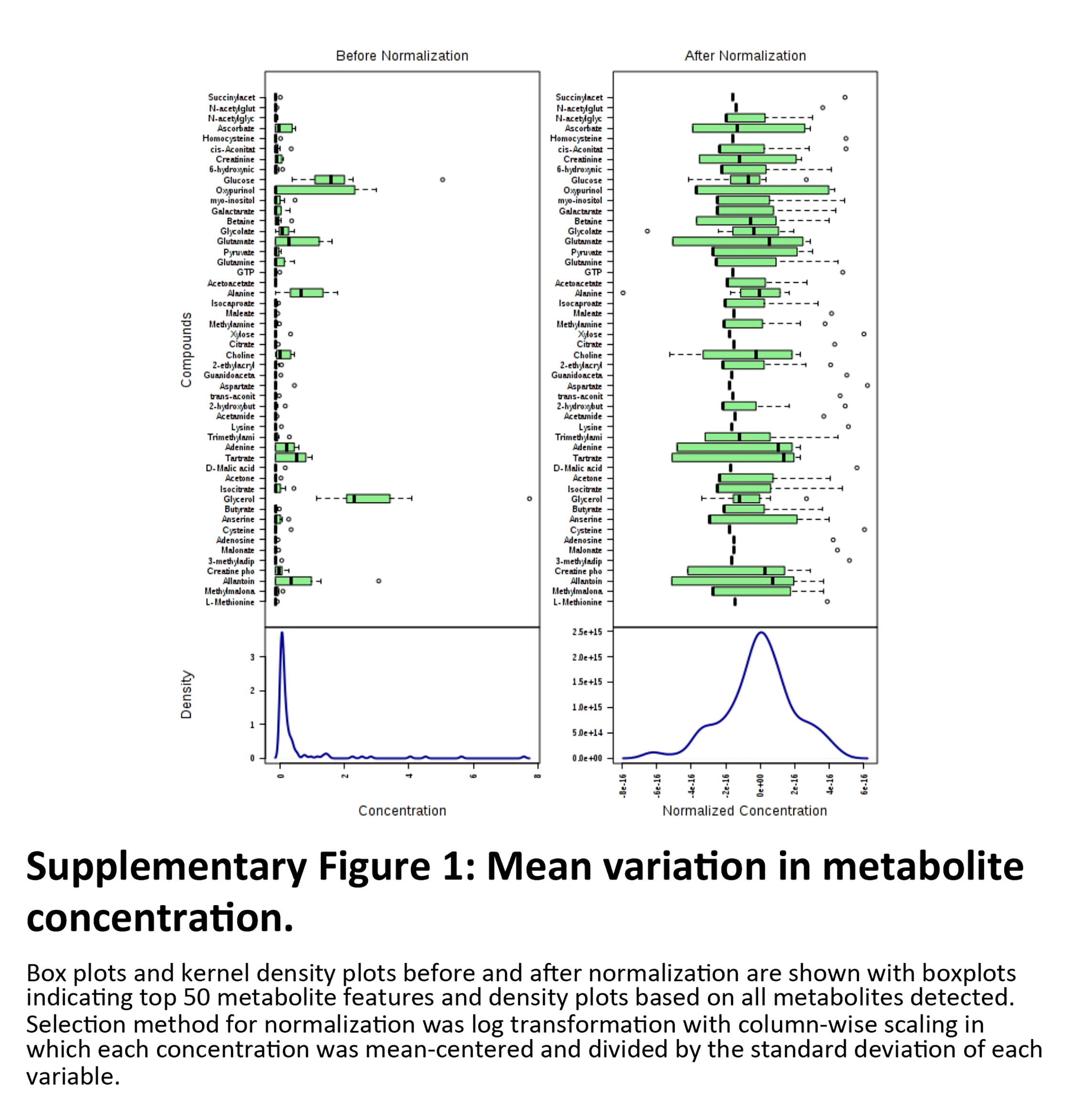

Supplement: S1 Fig — Box plots and kernel density plots before and after normalization are shown with boxplots indicating top 50 metabolite features and density plots based on all metabolites detected. Selection method for normalization was log transformation with column-wise scaling in which each concentration was mean-centered and divided by the standard deviation of each variable. (TIF) [file pone.0126735.s001.tif]

**Supplementary Table 1: 16S eubacterial primers used for 16S rRNA gene amplification.**


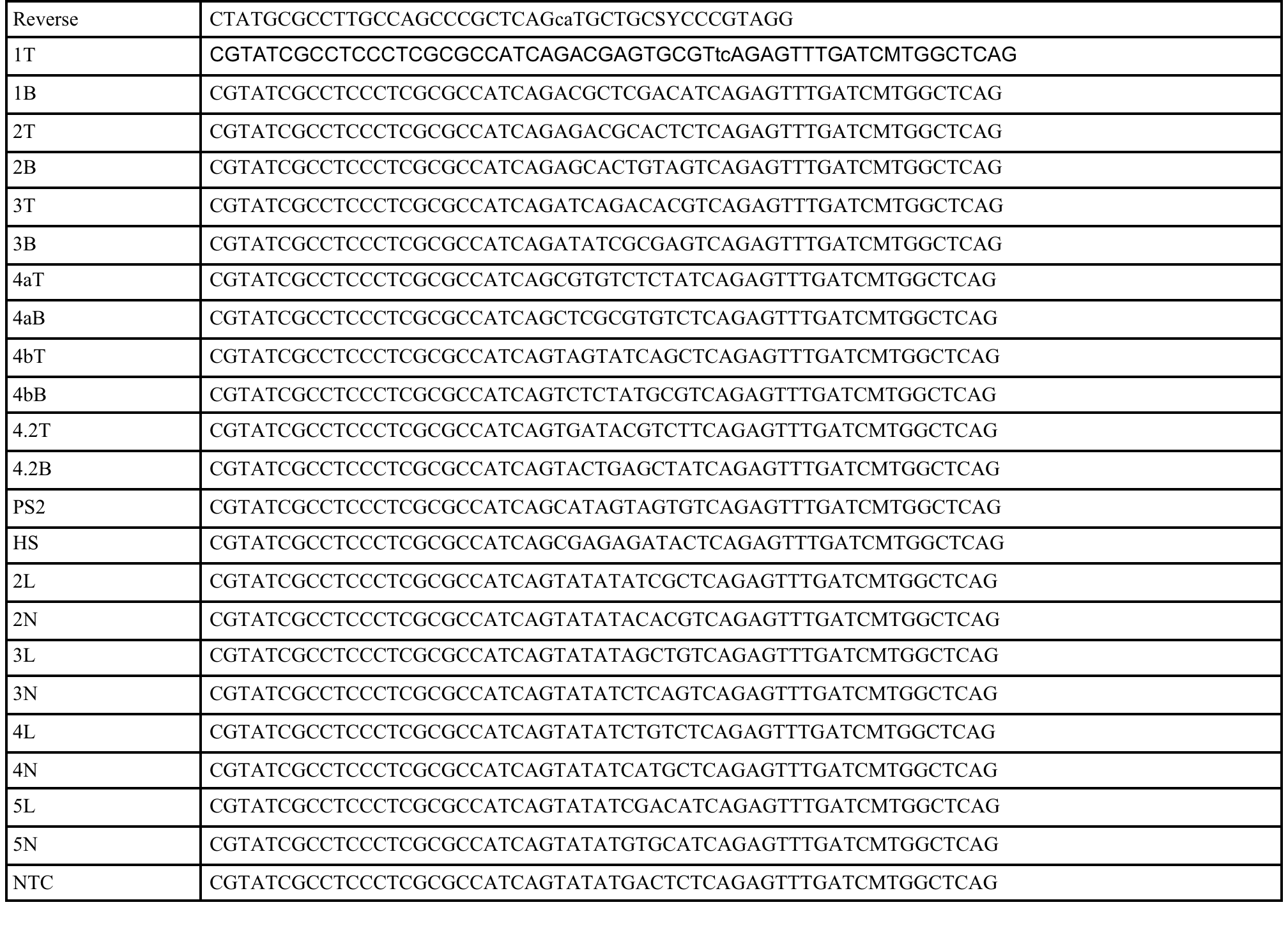

Supplement: S1 Table — (DOCX) [file pone.0126735.s002.docx]
