# Supplementary material for: Biochemical Association of Metabolic Profile and Microbiome in Chronic Pressure Ulcer Wounds
Source: PLoS One. 2015 May 15;10(5):e0126735. doi: 10.1371/journal.pone.0126735 (PMC4433261; doi:10.1371/journal.pone.0126735)
Supplement: S2 Table — (DOCX) [file pone.0126735.s003.docx]

**S2 Table. Relative abundance (%) of bacterial phyla in chronic pressure ulcers.**

|  |  | **Top of Biopsy** | | | |  | **Bottom of Biopsy** | | | |
| --- | --- | --- | --- | --- | --- | --- | --- | --- | --- | --- |
|  |  | **1T** | **2T** | **3T** | **4T** |  | **1B** | **2B** | **3B** | **4B** |
| **Phylum** | **Firmicutes** | 81.8 | 95.7 | 72.4 | 51.3 |  | 71.4 | 92.6 | 75.3 | 41.4 |
|  | **Proteobacteria** | 5.0 | 3.3 | 1.6 | 29.3 |  | 8.6 | 5.0 | 3.5 | 31.5 |
|  | **Actinobacteria** | 10.9 | 0.1 | 12.8 | 2.7 |  | 13.4 | 0.5 | 10.4 | 7.4 |
|  | **Bacteroidete** | 1.9 | 0.3 | 3.0 | 11.5 |  | 3.9 | 0.8 | 4.5 | 13.3 |
|  | **Cyanobacteria** | 0.2 | 0.0 | 0.2 | 0.0 |  | 0.1 | 0.0 | 0.3 | 0.9 |
|  | **Fusobacteria** | 0.0 | 0.0 | 9.5 | 0.0 |  | 0.0 | 0.0 | 4.9 | 2.6 |
|  | **Synergistetes** | 0.0 | 0.0 | 0.0 | 0.0 |  | 0.1 | 0.0 | 0.0 | 0.0 |
|  | **Other Bacteria** | 0.2 | 0.6 | 0.6 | 5.3 |  | 2.1 | 0.8 | 1.0 | 3.3 |
|  | **Unclassified** | 0.1 | 0.0 | 0.0 | 0.0 |  | 0.3 | 0.3 | 0.0 | 0.0 |
