# Supplementary material for: Biochemical Association of Metabolic Profile and Microbiome in Chronic Pressure Ulcer Wounds
Source: PLoS One. 2015 May 15;10(5):e0126735. doi: 10.1371/journal.pone.0126735 (PMC4433261; doi:10.1371/journal.pone.0126735)
Supplement: S4 Table — (DOCX) [file pone.0126735.s005.docx]

| **Supplementary Table 4: Factor Loadings for 2D PCA** | | | | | | | |
| --- | --- | --- | --- | --- | --- | --- | --- |
|  |  |  |  |  |  |  |  |
|  | **PC1** | **PC2** | **PC3** | **PC4** | **PC5** | **PC6** | **PC7** |
| **Actinobacteria** | 0.133 | 0.918 | 0.269 | 0.023 | 0.257 | -0.006 | 0.001 |
| **Bacteroidete** | 0.977 | -0.136 | 0.045 | -0.138 | 0.007 | 0.073 | 0.001 |
| **Cyanobacteria** | 0.763 | 0.294 | -0.197 | 0.531 | -0.103 | -0.001 | 0.000 |
| **Firmicutes** | -0.976 | -0.088 | -0.101 | 0.168 | -0.037 | 0.015 | 0.003 |
| **Fusobacteria** | 0.109 | 0.760 | -0.546 | -0.277 | -0.189 | -0.010 | 0.001 |
| **Proteobacteria** | 0.904 | -0.395 | 0.126 | -0.089 | 0.004 | -0.060 | 0.002 |
| **Synergistetes** | -0.088 | 0.294 | 0.928 | -0.020 | -0.213 | 0.002 | 0.000 |
| ^a^Threshold of significance set at a correlation coefficient of >0.400 | | | | | | | |
