# Supplementary material for: Biochemical Association of Metabolic Profile and Microbiome in Chronic Pressure Ulcer Wounds
Source: PLoS One. 2015 May 15;10(5):e0126735. doi: 10.1371/journal.pone.0126735 (PMC4433261; doi:10.1371/journal.pone.0126735)
Supplement: S6 Table — (DOCX) [file pone.0126735.s007.docx]

**Supplementary Table 6: Results table summarizing the metabolite set enrichment analysis (MSEA) ranked by *P* value (*p≤*0.1).**

| **Pathway** | **Total Metabolites** | **Metabolites Profiled** | **Holm P Value** | **FDR** |
| --- | --- | --- | --- | --- |
| **Protein Biosynthesis** | **19** | **11** | **1.0e^-^5** | **9.0e^-^4** |
| **Glycine, Serine, & Threonine Metabolism** | **26** | **11** | **5.0e^-^4** | **0.01** |
| **Ammonia Recycling** | **18** | **6** | **0.03** | **1.0** |
| **Citric Acid Cycle (TCA)** | **23** | **7** | **0.04** | **1.0** |
| **Betaine Metabolism** | **10** | **4** | **0.04** | **1.0** |
| **Methionine Metabolism** | **24** | **7** | **0.05** | **1.0** |
| **Urea Cycle** | **20** | **5** | **0.1** | **1.0** |
| **Arginine & Proline Metabolism** | **26** | **6** | **0.1** | **1.0** |
